# Nuclear dynamics of influenza A virus ribonucleoproteins revealed by live-cell imaging studies

**DOI:** 10.1016/j.virol.2009.08.015

**Published:** 2009-11-10

**Authors:** Eva M. Loucaides, Johann C. von Kirchbach, Ágnes Foeglein, Jane Sharps, Ervin Fodor, Paul Digard

**Affiliations:** a Division of Virology, Department of Pathology, University of Cambridge, Tennis Court Road, Cambridge CB2 1QP, UK; b Department of Applied Mathematics and Theoretical Physics, Centre for Mathematical Sciences, University of Cambridge, Cambridge CB3 0WA, UK; c Sir William Dunn School of Pathology, University of Oxford, South Park Road, Oxford, OX1 3RE, UK

**Keywords:** Influenza, Polymerase, FRAP, Ribonucleoprotein

## Abstract

The negative sense RNA genome of influenza A virus is transcribed and replicated in the nuclei of infected cells by the viral RNA polymerase. Only four viral polypeptides are required but multiple cellular components are potentially involved. We used fluorescence recovery after photobleaching (FRAP) to characterise the dynamics of GFP-tagged viral ribonucleoprotein (RNP) components in living cells. The nucleoprotein (NP) displayed very slow mobility that significantly increased on formation of transcriptionally active RNPs. Conversely, single or dimeric polymerase subunits showed fast nuclear dynamics that decreased upon formation of heterotrimers, suggesting increased interaction of the full polymerase complex with a relatively immobile cellular component(s). Treatment with inhibitors of cellular transcription indicated that in part, this reflected an interaction with cellular RNA polymerase II. Analysis of mutated influenza virus polymerase complexes further suggested that this was through an interaction between PB2 and RNA Pol II separate from PB2 cap-binding activity.

## Introduction

Influenza A virus ribonucleoprotein (RNP) particles contain all the viral components essential and sufficient for transcription and replication of the viral genome: one copy of the heterotrimeric RNA-dependent RNA polymerase formed by PA, PB1 and PB2 (3P), and the genomic vRNA wrapped around oligomerised nucleoprotein (NP) (Huang et al., 1990; Portela and Digard, 2002). Viral RNA synthesis occurs in the nucleus of infected cells (Herz et al., 1981; Jackson et al., 1982), where the viral polymerase transcribes the vRNA segments to generate mRNAs and also replicates the vRNAs via a complementary positive sense cRNA intermediate. Multiple host nuclear functions are parasitized during these processes, requiring an extensive interplay between viral and host components that is far from fully understood. Viral mRNA synthesis depends on RNA polymerase II (Pol II) to supply mRNA cap structures that are recycled from cellular pre-mRNAs as primers for viral transcription (Elton et al., 2006). It also requires Pol II activity for nuclear export of a subset of viral messages (Amorim et al., 2007; Vogel et al., 1994; Wang et al., 2008), as well as the splicing apparatus for processing of two viral mRNAs (Elton et al., 2006). Consistent with this, the 3P viral polymerase complex interacts with the large subunit of Pol II (Engelhardt et al., 2005; Mayer et al., 2007; Rameix-Welti et al., 2009) although whether this is to the benefit of the virus and/or the detriment of Pol II function remains to be determined (Chan et al., 2006; Rodriguez et al., 2007). A variety of other host derived nuclear interaction partners of the viral RNP components have also been defined (Amorim and Digard, 2006; Engelhardt and Fodor, 2006; Josset et al., 2008). Some of these cellular proteins are postulated to have accessory or inhibitory functions in trafficking of RNP and RNP components, viral transcription, and genome replication, but in many cases, the interaction is of unknown functional significance.

To further understand the interplay between influenza virus and the host cell we analysed the dynamic properties of viral RNP components in their authentic nuclear environment. Fluorescent Recovery After Photobleaching (FRAP) experiments were carried out on live-cells expressing recombinant green fluorescent protein (GFP)-tagged viral proteins. We find that the intranuclear dynamics of the influenza A virus RNA synthesis machinery is strongly influenced by the assembly state of its sub-components and the activity of host Pol II.

## Results

### Nuclear dynamics of recombinant influenza RNPs and their components

**Fig. 1:**
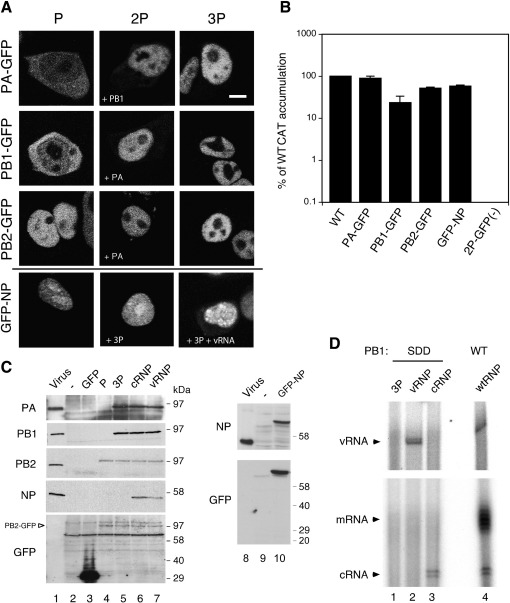
Reconstitution of recombinant GFP-tagged influenza RNPs in 293T cells. (A) Living cells expressing GFP-tagged RNP subunits alone or in combinations as indicated (2P; polymerase protein pairs, as labelled, 3P; the full polymerase complex) were analysed directly for GFP expression by confocal microscopy. Scale bar 5 μm. (B) Cells were transfected with plasmids expressing a synthetic vRNA encoding CAT and three (2P-GFP) or four of the RNP polypeptides, either all untagged (WT) or with the indicated one tagged with GFP. Three days post-transfection cells were analysed for CAT accumulation by ELISA. The mean and range from two independent experiments is plotted relative to the amount produced by WT RNPs. (C, D) Cells were transfected with plasmids expressing the indicated polypeptides (P, PB2-GFP; 3P, PB2-GFP, PA and PB1; cRNP and vRNP, PB2-GFP, PA and PB1 SDD along with NP and either a cRNA or vRNA containing a CAT gene respectively) or left untransfected (−). 24 h post-transfection (C) cell lysates and samples of purified virus (lane 1, as a marker) were analysed by SDS-PAGE and western blotting with antisera against PA, PB1, PB2, NP and GFP; (D) total RNA was extracted and analysed by primer extension using radiolabelled oligonucleotides specific for negative (top panel) or positive sense (lower panel) CAT transcripts. Radiolabelled products were separated by urea-PAGE and detected by autoradiography. Primer extension products resulting from the indicated CAT RNA species are labelled.

To investigate the dynamics of viral RNP components in FRAP experiments, plasmids expressing C-terminally GFP-tagged polymerase proteins (Fodor and Smith, 2004) or an N-terminally GFP-tagged NP molecule were employed and the system for recreating recombinant RNPs validated. First, we analysed the localisation of GFP-tagged polymerase subunits on their own or in dimer (2P) or trimer (3P) combinations with the other untagged P subunits in living 293T cells. All GFP-tagged subunits expressed in the full polymerase (3P) context localised to the nucleus (Fig. 1A). Individually expressed PB2-GFP also accumulated in the nucleus while PA-GFP and PB1-GFP were only efficiently imported in the presence of the other (Fig. 1A). This confirms data previously obtained on fixed cells indicating that PA and PB1 undergo nuclear import as a dimer (Fodor and Smith, 2004). GFP-NP also localised efficiently to the nucleus whether expressed alone or with a model vRNA and/or 3P (Fig. 1A, lower panels). Next, cells were multiply transfected with plasmids expressing a model cRNA segment containing a chloramphenicol acetyl-transferase (CAT) gene and various combinations of tagged and untagged polymerase and NP proteins to recreate functional RNPs (Fodor et al., 2002; Huang et al., 1990; Mullin et al., 2004). Consistent with a previous study (Fodor and Smith, 2004), analysis of CAT accumulation confirmed the transcriptional competence of the derivatised P and NP proteins. Replacement of any single RNP polypeptide with its GFP-tagged counterpart resulted in between ~ 25% and 95% of the activity seen with an untagged RNP, and in all cases activity was several hundred fold higher than the background obtained in the absence of PB1 (Fig. 1B). Cell lysates were also analysed by SDS-PAGE and Western blotting. All three polymerase subunits were detected in similar quantities whether in the 3P or RNP context, while NP was present in RNP-transfected samples (Fig. 1C, lanes 3–7, 10). Anti-GFP detected both GFP and PB2-GFP at the expected sizes with no obvious GFP-containing degradation products in the latter (Fig. 1B, lanes 3 and 4–7), confirming expression of a full-length GFP-tagged polymerase subunit alone and in combination with other viral proteins. Similarly, analysis of GFP-NP transfected samples confirmed expression of a polypeptide of the expected size that reacted with both anti-NP and anti-GFP with no obvious degradation products (Fig. 1C, lanes 8–10). The presence of viral RNAs in transfected cells was verified using reverse transcription primer extension analysis. In cells transfected with the viral polymerase alone no viral RNA species could be detected (Fig. 1D, lane 1). In the presence of replication competent RNPs, vRNA as well as m- and cRNA were detected, indicating active viral transcription and replication (Fig. 1D, lane 4). When WT PB1 was substituted with a PB1-SDD active-site mutant (Vreede et al., 2004) model vRNPs or cRNPs, according to the polarity of the reporter segment employed, were obtained. In such samples, only RNA of the corresponding input polarity was detected (Fig. 1D, lanes 2–3), confirming encapsidation and protection of Pol I-transcribed RNA into transcriptionally inert RNPs (Vreede et al., 2004). Thus overall, the recombinant system used here successfully reconstituted GFP-tagged influenza virus RNPs.

**Fig. 2:**
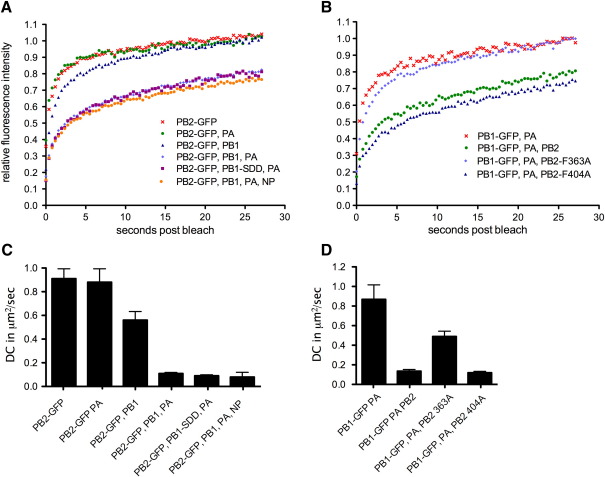
Nuclear mobility of viral polymerase proteins. 293T cells were transfected with plasmids expressing (A) PB2-GFP alone or (B) PB1-GFP and PA in combinations with other polymerase proteins and NP as indicated. 24 h post-transfection fluorescent cells were analysed by FRAP. The average recovery kinetics are plotted. (C, D) The mean DC ± SEM for each transfection combination are plotted. See Table 1 for information on the number of repetitions and statistical analyses.

**Fig. 3:**
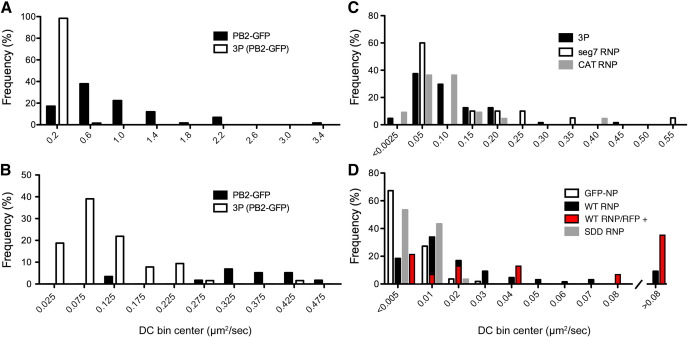
Distribution of DC values from individual cells. Histograms of DC values from cells transfected with the indicated plasmids are shown. Data are plotted on bin centres as the % of the total for each category. The same data are plotted in (A) and (B) but with a smaller bin size in (B) to resolve the spread of 3P DC values.

Next, we employed FRAP to analyse the nuclear dynamics of influenza A virus RNP components, pursuing a strategy in which RNPs were built up incrementally, allowing comparison of the mobility of the various sub-assemblies with the final functional entity. Accordingly, 293T cells were transfected with plasmid mixtures expressing individual GFP-tagged polymerase subunits alone, in combination with a second untagged polymerase subunit, or with both other subunits to reconstitute the full heterotrimeric polymerase. PB2-GFP displayed very rapid recovery kinetics and fully recovered to initial fluorescence intensity indicating the virtual absence of immobile molecules (Fig. 2A). To quantify P protein nuclear dynamics, mean time to half recovery (*t*_1/2_) and diffusion coefficient (DC) values were calculated from the fluorescence recovery data for individual cells, utilising multiple repeat experiments. (Fig. 2C, Table 1). A degree of cell-to-cell variability was seen among the estimates for the DC and the half recovery time for each particular setting, but when the distributions of individual DC values were plotted on histograms most of them formed a bell-shaped distribution (Fig. 3A). This validates the idea of a typical time for recovery for that setting and also the approach of estimating that time by taking an average of the estimates for each individual cell. These data confirmed the fast nuclear dynamics of PB2 alone with an average *t*_1/2_ value of around 1 s and a DC of nearly 1 μm^2^/s (Fig. 2C, Table 1). Co-expression of PA did not significantly alter PB2-GFP dynamics (Figs. 2A, C, Table 1), consistent with the weak interaction between PB2 and PA in the absence of PB1 (Digard et al., 1989; Hemerka et al., 2009). Expressing PB2-GFP in combination with PB1 caused a slight reduction (less than 2-fold; Fig. 2C, Table 1) in the rate of fluorescence recovery, although recovery still reached 100% of initial fluorescence intensity (Fig. 2A). However, a highly statistically significant (as assessed by *t*-test) near 10-fold decrease in DC was observed upon expression of the full 3P complex (Fig. 2C, Table 1). Fluorescence recovery was also incomplete, suggesting a fraction of the influenza polymerase was now static within the nucleus during the time span of the experiment (Fig. 2A). When the spread of DC values from individual cells was considered, they again formed a bell-shaped distribution that furthermore, showed only minimal overlap with the values obtained from cells expressing only PB2-GFP (Fig. 3B). Thus although the FRAP technique does not distinguish between ‘free’ PB2-GFP molecules and PB2-GFP incorporated into a 3P complex in any one cell, the distinctly different distributions of DCs from single cells expressing either PB2-GFP alone or in the 3P context suggests that the majority of the latter cells contained a significant population of PB2-GFP in the form of the polymerase complex. Reliable data concerning the nuclear mobility of either PA-GFP or PB1-GFP expressed alone could not be generated because of their inefficient nuclear import as monomers (Fig. 1A). However, both tagged forms of the PB1-PA dimer showed similarly fast nuclear dynamics to PB2-GFP that again in both cases, dropped dramatically on formation of the full 3P complex (Figs. 2B, D, Table 1). Overall, depending on which GFP-tagged polymerase subunit was employed, between 2- and 10-fold increases in *t*_1/2_ and 3- to 10-fold reductions in DC were observed on formation of the 3P complex, with the smallest alterations occurring with a labelled PA subunit. *t*-test analysis showed these changes in *t*_1/2_ and DC upon full polymerase assembly to be highly significant (Table 1). Thus assembly of the full 3P complex results in the appearance of an immobile fraction of the polymerase and markedly lower overall intranuclear dynamics.

**Fig. 4:**
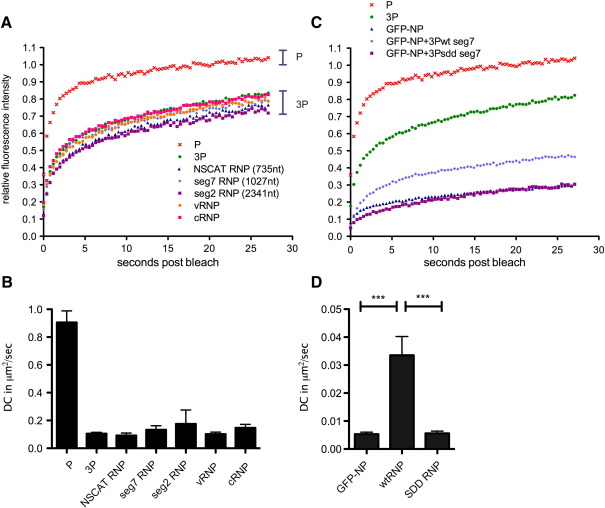
Nuclear mobility of viral RNPs and NP. (A, B) 293T cells were transfected with plasmids expressing PB2-GFP alone (P) or in combinations with the other polymerase proteins (3P), NP and model genome segments as indicated or (C, D) GFP-NP alone, in the context of a WT segment 7 RNP (+3Pwt seg7) or in the context of a replication incompetent segment 7 RNP (+3Psdd seg7). 24 h post-transfection fluorescent cells were analysed by FRAP and (A, C) recovery kinetics were plotted, for comparison, alongside recovery curves for PB2-GFP alone and in the heterotrimeric polymerase context. (B, D) The mean DC ± SEM for each transfection combination are plotted. Asterisks and brackets indicate the probability (⁎⁎⁎ *p* < 0.001; Student's two-tailed *t*-test, assuming equal variance) of the compared data being equal.

Next, the effect of RNP formation on polymerase nuclear dynamics was examined. NP binds to both PB1 and PB2 in the absence of vRNA (Biswas et al., 1998; Medcalf et al., 1999), but its co-expression with PB1-GFP tagged 3P in the absence of the viral genome did not alter polymerase recovery rates (Figs. 2A, C). Similarly, when a plasmid expressing the model NSCAT vRNA segment was additionally transfected to permit the formation of full RNPs, no significant alteration to polymerase dynamics was seen (Figs. 4A, B). RNP size depends on segment length (reflecting the stoichiometric requirement for encapsidating NP at approximately 1 NP:24 nucleotides [Portela and Digard, 2002]), with predicted molecular weights ranging from approximately 2 MDa for the NSCAT RNP to over 6 MDa for the two largest viral segments. However, when RNPs were recreated with either of segments 2 or 7, no significant alteration to polymerase mobility was seen. The small variations that were seen showed no correlation with RNP size (Fig. 4A), nor were the dynamics of the vRNPs statistically significantly different from the dynamics of the isolated polymerase complex (Fig. 4B and data not shown). Primer extension analysis of RNA isolated from the transfected cells indicated successful transcription and replication of the input vRNA molecules (data not shown), confirming successful reconstitution of RNPs in a least a subset of the transfected cells. However, no shift in the distribution of even a minority of the individual cell DC values between 3P and RNP transfected samples was evident (Fig. 3C). Thus the formation of active RNPs does not influence the overall dynamics of the viral polymerase in this system. However, vRNA and cRNA undergo differential trafficking in infected cells with the former but not the latter molecules being exported to the cytoplasm (Shapiro et al., 1987; Tchatalbachev et al., 2001). The reason why cRNPs are retained in the nucleus is not known but one hypothesis is that it results from a high affinity interaction with a static component of the nucleus. Thus it was possible that the intranuclear dynamics of RNPs formed around the two polarities of viral genomic RNA might differ. To test this, we reconstituted transcriptionally inert RNPs (tagged with PB2-GFP) using the PB1-SDD mutant and plasmids that expressed either vRNA or cRNA polarity NSCAT molecules to produce either vRNPs or cRNPs. Primer extension analysis of RNA from the transfected cells again confirmed expression of the expected species (Fig. 1C). However, FRAP measurements produced recovery curves and DC values that were essentially indistinguishable from that of the 3P polymerase complex, thus not supporting the hypothesis that segment polarity affects the nuclear dynamics of influenza RNPs (Figs. 4A, B).

Since formation of RNP complexes does not affect the nuclear dynamics of the influenza polymerase we next investigated the effect of RNP formation from the perspective of NP. NP is the major protein component of viral RNPs but is also present as free molecules in infected cells (Beaton and Krug, 1986; Rees and Dimmock, 1982). Thus the mobility of GFP-NP in the absence or presence of other RNP components was examined. In FRAP analysis of cells expressing recombinant GFP-NP alone, fluorescence recovered very slowly post-bleach and only to about 30% of initial fluorescence intensity (Fig. 4C). Therefore, GFP-NP exhibits markedly slower nuclear dynamics than any individual polymerase subunit or even the full polymerase complex. However, co-expression of 3P and segment 7, thus allowing RNPs to form, lead to a pronounced increase in recovery rates, with recovery to nearly 50% of initial fluorescence intensity and a significant 6-fold increase in the DC (Figs. 4C, D). Examination of the DC values from individual cells showed that this increase in average DC reflected a relatively small increase in values from the majority of cells coupled with the appearance of a population of cells (around 20% of the total) with large increases in DC of 10-fold or greater (Fig. 3D). To examine whether these findings held true in the context of a transcriptionally inert polymerase, PB1 was replaced with PB1-SDD leaving the other RNP components unchanged. In this context, GFP-NP displayed slow average dynamics, similar to when expressed on its own and significantly different to the higher mobility it adopted in the context of active RNPs (Figs. 4C, D). Examination of the DC values from individual cells showed that all cells analysed contained slow-moving NP (Fig. 3D), in contrast to when active RNPs were reconstituted. The same effects were seen when NSCAT vRNA was used as the model segment (data not shown). To further probe the association between NP dynamics and viral RNA synthesis, cells containing transcriptionally active RNPs were marked by utilising a vRNA encoding RFP. The majority (though not all) of these cells harboured highly mobile populations of GFP-NP (Fig. 3D). Thus, formation of transcriptionally active RNPs affects the intranuclear dynamics of NP, but unexpectedly, by increasing its mobility.

### A role for cellular transcription in nuclear dynamics of the viral polymerase

**Fig. 5:**
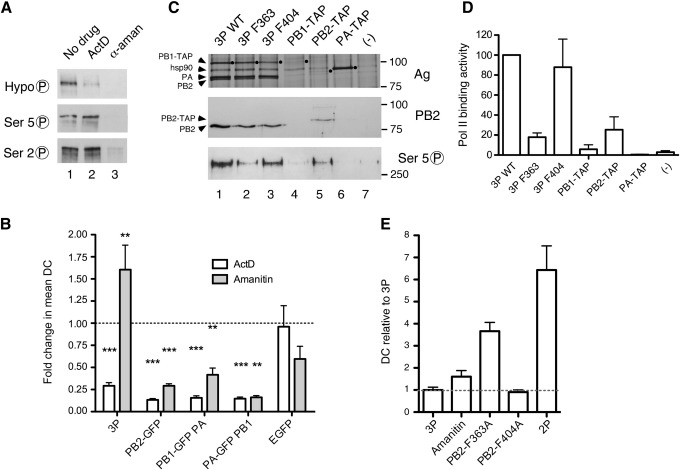
Role of cellular transcription in the nuclear dynamics of the viral polymerase. (A) 293T cells were mock-treated (−), treated with 5 μg/ml ActD for 1 h or with 5 μg/ml α-amanitin for 9 h, lysed and analysed by SDS-PAGE and Western blotting with sera specific for modified forms of the CTD of RNA Pol II as indicated. (B) 293T cells were transfected with expression vectors for the indicated GFP-tagged viral polymerase proteins (3P complex tagged with PB2-GFP) or GFP, 24 h post transfection treated or mock treated with RNA Pol II inhibitors as in (A) and fluorescent cells then analysed by FRAP. The fold change in mean DCs of the drug-treated samples relative to the corresponding untreated samples are plotted. A Student's two-tailed *t*-test, assuming equal variance, was used to compare DCs of drug-treated and untreated samples and returned *p*-values (⁎⁎*p* < 0.01, ⁎⁎⁎*p* < 0.001) describing the probability of the compared data being equal. (C) 293T cells were transfected with expression vectors for the indicated TAP-tagged viral polymerase proteins (3P complex tagged with PB1-TAP) and 42 h later cell extracts fractionated by IgG sepharose chromatography followed by SDS-PAGE and (top panel) silver staining; migration of TAP-tagged subunits indicated by black spots, (middle panel) western blotting for PB2 or (lower panel) serine 5 phosphorylated Pol II. (D) Bound Pol II from three independent experiments was quantified by densitometry and plotted as the mean ± SEM relative to WT 3P (100%). (E) The fold change in mean DC values of the indicated samples relative to WT 3P are plotted. The 2P value is from PB1-GFP + PA.

Evidence for an interaction between the influenza polymerase and cellular Pol II (Engelhardt and Fodor, 2006; Mayer et al., 2007; Rameix-Welti et al., 2009) as well as the dependence of viral gene expression on functionally and spatially interlinked cellular transcription and mRNA processing machinery (Amorim et al., 2007; Braam et al., 1983) suggest the hypothesis that the cellular transcriptosome influences the nuclear dynamics of the viral polymerase. To test this, cells transfected for FRAP analysis were treated with two inhibitors of cellular RNA polymerase II: actinomycin D (ActD) and α-amanitin. To confirm activity of the drug treatments, cell lysates were analysed by SDS-PAGE and western blotting using sera to various phosphorylated forms of the C-terminal repeat domain (CTD) of the large subunit of Pol II. As expected (Casse et al., 1999), ActD treatment increased levels of serine 2- and serine 5–phosphorylated Pol II while concomitantly, levels of unmodified serine 2 decreased (Fig. 5A). Also as expected (Casse et al., 1999; Nguyen et al., 1996), α-amanitin treatment drastically reduced detection of all forms of Pol II with only low levels of serine 2-phosphorylated RNA polymerase II detectable in cells treated with the drug (Fig. 5A). When drug-treated cells were examined by FRAP, neither ActD nor α-amanitin treatment significantly altered the nuclear mobility of GFP (Fig. 5B; data plotted as fold change in DC in response to the drug treatment), suggesting that disruption of cellular transcription did not modify the nuclear environment sufficiently to affect the dynamics of an irrelevant protein. However, the full viral polymerase complex underwent a highly significant 5-fold reduction in mobility when cells were treated with ActD. α-Amanitin, on the other hand, significantly increased mobility of the 3P complex with a 1.6-fold increase in mean DC (Fig. 5B). This supports the hypothesis that cellular Pol II affects the intranuclear dynamics of the viral polymerase. Previous work has shown that a stable interaction between the Pol II CTD and viral polymerase requires all three viral P proteins to be present (Engelhardt et al., 2005). Therefore, we tested whether the effect of inhibiting cellular transcription was specific to 3P dynamics. Unexpectedly, the mobility of PB2-GFP and the GFP-tagged PA-PB1 dimer was significantly reduced 2- to 5-fold by both ActD and α-amanitin treatment (Fig. 5B). Thus, inhibition of the cellular transcriptosome by ActD DNA intercalation decreased mobility of all viral polymerase components tested, while degradation of Pol II due to α-amanitin treatment accelerated the dynamics of only the full viral polymerase; in contrast subassemblies of the P complex were rendered less mobile.

In addition to protein–protein contacts between the viral and cellular polymerases, there is also the possibility of protein–RNA bridges, the most obvious of which is via the mRNA cap-binding activity of PB2 (Elton et al., 2006). To test the contribution this interaction makes to the intranuclear dynamics of the viral polymerase, we reconstituted the 3P complex with either of two PB2 proteins containing mutations (F363A or F404A) known to block cap-binding activity (Fechter et al., 2003; Guilligay et al., 2008). Surprisingly, these two mutants behaved differently in the FRAP assay. A polymerase complex incorporating the PB2-F404A mutant behaved similarly to the WT trimer (Figs. 2B, D). In contrast, co-transfection of PB2-F363A caused only a slight reduction in the dynamics of the PB1-GFP:PA dimer (Figs. 2B, D, numerical values given in Table 1). The simplest explanation for the differing phenotypes of the two cap-binding mutants was that the F363A mutation affected the folding of the protein such that it no longer formed a stable complex with PB1 and PA. Therefore, to test 3P complex formation by the mutant PB2 polypeptides, cells were transfected with combinations of plasmids encoding tandem affinity purification (TAP) sequence-tagged variants of a single P protein in the presence or absence of the untagged partners. TAP-tagged and interacting proteins were then partially purified by IgG sepharose affinity chromatography (Fodor and Smith, 2004). Individually expressed TAP-tagged PA expressed well, but as before (Deng et al., 2005), PB1-TAP and PB2-TAP alone were recovered in relatively low amounts (Fig. 5C top panel, lanes 4–6). Comparison with material obtained from untransfected cells showed recovery of some background cellular proteins, including hsp90 (lane 7). However, samples purified from triply-transfected cells showed abundant quantities of all three P proteins, whether the complex was reconstituted with WT PB2 or either cap-binding mutant (lanes 1–3). Because PB2 and PA nearly co-migrate, levels of co-purified PB2 were further examined by western blotting. This confirmed that all three P protein complexes contained equivalent amounts of PB2 (Fig. 5C, middle panel). Therefore, as both PB2 mutants are known to be defective in cap-binding (Fechter et al., 2003; Guilligay et al., 2008) yet form similarly stable 3P complexes, we sought another explanation for their altered intranuclear dynamics. In light of the identified interaction between the influenza and cellular RNA Pol II complexes (Engelhardt et al., 2005; Mayer et al., 2007; Rameix-Welti et al., 2009) and the effect transcriptional poisons had on 3P dynamics (Fig. 5B), we tested if the PB2 mutations affected Pol II-binding by immunoblotting the TAP-purified influenza polymerase samples for co-purified RNA Pol II. As before (Engelhardt et al., 2005), only trace amounts of serine 5-phosphorylated Pol II were detectable in samples purified from mock transfected cells, whereas abundant quantities co-purified with the WT influenza polymerase (Fig. 5C lower panel, compare lanes 1 and 7). Similar amounts of Pol II co-purified with a 3P complex containing the PB2-F404A mutant but much reduced quantities associated with the trimer incorporating the F363A mutant (compare lanes 1, 2 and 3). When replicate experiments were quantified, binding activities of the WT and F404A mutants were equivalent but the F363A-containing complex associated with less than one-fifth the normal amount of Pol II (Fig. 5D). Thus the F404A mutation in PB2 which severely reduces cap-binding activity (Fechter et al., 2003; Guilligay et al., 2008) but does not affect Pol II binding displays normal intranuclear dynamics while mutant F363A with deficits in both cap- and pol II-binding activities shows much faster dynamics (Fig. 4D). This suggests that the interaction between PB2 and mRNA cap-structures is not a major determinant of polymerase mobility but provides further evidence for the importance of interactions with cellular Pol II. Furthermore, appreciable quantities of Pol II bound to PB2-TAP alone, while PB1-TAP also showed activity slightly above background (Fig. 4C, lanes 4 and 5, quantification in Fig. 5D). This contrasts with previous work in which only the 3P complex was able to detectably bind to the isolated Pol II CTD (Engelhardt et al., 2005) but is consistent with the altered FRAP mobility of PB2-GFP and GFP-tagged PB1-PA dimers after treatment with transcriptional inhibitors (Fig. 4B). Overall, this suggests that the influenza RNA polymerase may make additional contacts with other subunits and/or domains of the intact RNA Pol II.

## Discussion

We have established a FRAP system that has yielded insights into the nuclear dynamics of influenza virus RNPs in an authentic live-cell environment. To our knowledge, no such system has previously been described. Although it employs subsets of the normal viral genes expressed from plasmids, RNPs reconstituted with the tagged subunits were transcriptionally active (Figs. 1B, D) and thus their behaviour is likely to be a fair reflection of the behavior of authentic RNP components. We show that in a mammalian cell, RNP assembly state influences the mobility of both NP and the viral polymerase. The full 3P complex displays markedly slower mobility than single polymerase subunits or dimer combinations, while NP mobility significantly increases in response to active RNP formation. We also find that cellular RNA Pol II function is an important determinant of the dynamics of the viral polymerase.

The assembly state of the viral heterotrimeric polymerase strongly influences its nuclear dynamics in mammalian cells with the full trimer showing markedly reduced mobility compared to individual subunits or dimers. The relatively fast intranuclear diffusion of PB2 and PB1-PA is compatible with current data indicating the two sub-assemblies undergo separate nuclear import (Deng et al., 2005; Fodor and Smith, 2004; Naito et al., 2007); slow intranuclear dynamics of the sub-complexes might otherwise act as a kinetic barrier to formation of the full 3P polymerase complex. It is also worth considering reasons for the marked decrease in diffusion rate of the 3P complex compared to its individual components. One possibility is an increase in size; in simple terms, from ~ 85 kDa (any of the monomers) to ~ 170 kDa (dimer) to ~ 250 kDa (trimer). However, according to standard diffusion theory a mere three-fold increase in size of a soluble, globular protein complex cannot account for such marked reduced mobility (Lippincott-Schwartz et al., 2001). In addition, evidence suggests that at least some of the PB1-PA and PB2 molecules will be in complex with cellular proteins that are lost on formation of the viral heterotrimer (Deng et al., 2005; Fodor and Smith, 2004; Naito et al., 2007), further reducing the mass differential of the various complexes. While recombinant viral polymerase has been shown to be globular in appearance both in its soluble and RNP-associated form (Area et al., 2004; Torreira et al., 2007) it remains to be clarified whether, and to what extent, the influenza polymerase multimerizes. Sedimentation and co-precipitation analyses have provided evidence both for (Digard et al., 1989; Jorba et al., 2008) and against (Honda et al., 1990) polymerase aggregates (although the latter study examined virion-derived polymerase, which may be a special case). It is also worth noting that the EM image analysis method used to visualise the polymerase (Area et al., 2004; Torreira et al., 2007) is biased towards picking similar structures and would therefore filter out heterogeneous aggregates. Thus while an increase in complex size seems an unlikely explanation for the slow mobility of the full polymerase complex, it will nevertheless be worthwhile to clarify the extent of polymerase aggregation in the FRAP setting. Reduced recovery rates of photobleached polymerase trimers correlated with a substantial increase in an apparently immobile pool of 3P as the fluorescence signal never recovered to initial intensity levels. Overall therefore, we favour the hypothesis that the slow intranuclear mobility of the full viral polymerase complex results from a specific interaction between the trimer and a relatively immobile cellular component(s).

Bearing in mind the substantial increase in size from a polymerase trimer to a full RNP complex, it was surprising that no effect of RNP formation on polymerase dynamics could be detected. One possibility is that only a relatively small number of transfected cells successfully reconstituted active RNPs. However, this is inconsistent with the increase in GFP-NP mobility seen after RNP reconstitution, where an affect of RNP formation on the mobility of the tagged polypeptide was clearly demonstrable, both on the dynamics of the total population and at the level of single cells (Figs. 3D, 4C, D). Furthermore, control experiments examining the efficiency of plasmid-directed RNP formation (by fluorescent detection of the mRNA or protein product encoded by the vRNA reporter) indicated that between ~ 10% and 90% of cells expressing either NP or PA also contained active RNPs, depending on the reporter vRNA and detection method employed (data not shown). Nevertheless, no effect of RNP formation was seen on the mobility of the polymerase complex, whether in aggregate (Figs. 4A, B) or at the level of individual cells (Fig. 3C). Therefore it is possible that the polymerase-RNP interaction is highly dynamic and occurs fast enough to not mask the characteristic mobility of the polymerase heterotrimer. Recent work showed that the interaction of the polymerase complex with the 5′ end of vRNA and especially cRNA is indeed a dynamic process (Dalton et al., 2006). However, *in vitro* dissociation of the polymerase from its template was measurable over periods of minutes rather than the seconds of FRAP experiments, so it is not clear whether the two cases are necessarily comparable. Thus a more conservative (but not necessarily exclusive) explanation for the lack of effect of RNP formation on 3P dynamics is simply that the bulk of the polymerase is not RNP-associated in this system. This explanation does however parallel the situation in infected cells, where a pool of non-RNP associated polymerase complex exists (Akkina et al., 1987; Detjen et al., 1987).

In contrast to the behaviour of the polymerase, the nuclear mobility of NP, the major protein component of RNPs, was strongly influenced by RNP assembly state. Recombinant GFP-NP dynamics were very slow in the absence of other viral proteins, but were significantly faster in the presence of the other RNP components, as long as they were transcriptionally competent. The failure to increase NP mobility when transcriptionally inert RNPs were reconstituted could perhaps be attributed to lower levels of model genome segments available for NP to interact with, in the absence of amplification of input RNA segments by the polymerase. However, primer extension analysis detected significant levels of c- and vRNA transcribed from the input plasmids that were not drastically increased upon WT RNP formation (Fig. 1C). Thus NP is more mobile when associated with active RNPs than when co-expressed with the components of replication incompetent RNPs. One possible explanation for this is that the presence of active viral RNPs alters the nuclear environment in such a way as to alter the dynamics of GFP-NP monomers. However, we prefer the hypothesis that active transcription of RNPs displaces NP bound to the template RNA. This mechanism could provide a solution to the problem of steric hindrance that must otherwise be faced by a polymerase that remains bound to the 5′-end of vRNA as well as the internal region being copied during mRNA transcription (Elton et al., 2006; Pritlove et al., 1998). A complete release of NP monomers from the RNP structure during transcription contrasts with a model proposed for transcription of non-segmented negative strand viruses which suggests that the transcribing polymerase gains access to the encapsidated template due to a conformational change in the N protein that locally opens the structure without disrupting the polymeric N protein backbone of the RNP (Albertini et al., 2008). Nevertheless, orthomyxovirus RNPs differ fundamentally from non-segmented virus RNPs in other aspects of their structure (Klumpp et al., 1997; Pons et al., 1969) so such a difference is not implausible. If transcription does displace NP from the RNP, thereby causing an overall increase in the mobility of the protein pool, recruitment of the soluble protein to the structure(s) responsible for the very slow dynamics of the non-RNP form must be relatively slow. An NP mutant (R416A) with a primary defect in oligomerisation (Elton et al., 1999a; Elton et al., 1999b; Ye et al., 2006) displayed much faster nuclear dynamics (data not shown), suggesting that the ability to self-associate may be key to the low nuclear mobility of GFP-NP. While this might thus be a size effect alone, it is also possible that multimerisation is essential for a high avidity interaction of NP with an insoluble nuclear component. For example NP is known to interact with chromatin components (Bukrinskaya et al., 1979; Garcia-Robles et al., 2005).

Use of drugs that inhibit cellular transcription confirmed that the cellular Pol II transcriptosome influences the nuclear dynamics of the viral polymerase, as did the correlation between the abnormally fast diffusion of a 3P complex containing the PB2-F363A mutant with a defect in Pol II-binding. However, Pol II cannot be the sole determinant of viral polymerase mobility because although its removal through α-amanitin treatment increased the DC of the influenza polymerase, it was not to the level of single or dimeric polymerase polypeptides, or even to that of the PB2-F363A mutant (Fig. 4E), all of which retain some ability to interact with Pol II (Fig. 4D). In similar vein, we have also considered the hypothesis that the sharp drop in DC seen on assembly of the full heterotrimeric influenza polymerase results from its interaction with RNA Pol II, as initial work found that only the 3P complex bound to the CTD of Pol II stably enough to be detected by biochemical means (Engelhardt et al., 2005). However, treatment with ActD and α-amanitin altered the mobility of not only the 3P complex but also PB2-GFP alone and the PB1-PA dimer (Fig. 4B). Moreover, when binding of influenza P proteins to intact RNA Pol II (as opposed to the isolated CTD; (Engelhardt et al., 2005)) was examined, a clear interaction with isolated PB2 was seen, as well as possibly weak binding by PB1 (Figs. 4C, D). The difference in expression levels between individually and co-expressed PB2 (and PB1) makes it difficult to compare the relative binding strengths of single and trimeric P proteins, but it seems likely that PB2 contributes significantly to the interaction with Pol II, possibly via the cap-binding domain. Overall, the FRAP analyses of polymerase mobility are likely to report a summation of multiple interactions, some too weak to be easily detected by biochemical means. We suspect that the complex effects of transcriptional poisons on the dynamics of sub-assemblies of the viral polymerase reflects this, as well as potential indirect effects on the nuclear environment resulting from the inhibition of cellular mRNA synthesis.

Overall, the live-cell imaging and FRAP system described here has proven useful to further understand the intracellular dynamics of the four viral polypeptides minimally required for transcription and replication of the virus genome and their interactions with the cellular transcription machinery. Further work will explore its potential to inform on the interactions of other viral polypeptides that while not essential for viral RNA synthesis, nevertheless interact with RNPs (Bullido et al., 2001; Kuo and Krug, 2009; Marion et al., 1997; Mazur et al., 2008; Robb et al., 2009; Wise et al., 2009) as well as with interactions with cellular proteins potentially involved in determining influenza virus host range (Naffakh et al., 2008).

## Materials and methods

### Cell culture, virus, plasmids and drugs

Human embryonic kidney 293T cells were cultured and transfected with plasmids as described (Amorim et al., 2007). Plasmids expressing untagged A/PR/8/34 (PR8) and GFP-tagged A/WSN/33 (WSN) PB2, -PB1, -PA and PR8 GFP-NP proteins as well as pPol-I(+)NS.CAT and pPol-I(−)NS.CAT have previously been described (Fodor and Smith, 2004; Mullin et al., 2004). Plasmids encoding the WSN PB2-F363A and –F404A mutants are described in (Fechter et al., 2003). Plasmid pcDNA-PB1-SDD was constructed by site-directed mutagenesis using the primers 5′-TCAATCCTCTGCAGCTTTTGCTCTGA and 5′-TCAGAGCAAAAGCTGCAGAGGATTGA generating a double mutation in the conserved SDD motif of the polymerase active site of PB1 (PB1-D445A/D446A). Plasmids pPolI-seg1 and pPolI-seg7 that express PR8 vRNA segments 1 and 7 respectively were the generous gift of Professor Ron Fouchier (de Wit et al., 2004). pPOLI-DsRed was generated by replacing the chloramphenicol acetyltransferase (CAT) open reading frame (ORF) of the pPOLI-CAT-RT plasmid (Pleschka et al., 1996) with red fluorescent protein (dsRed) ORF amplified by PCR using pDsRed1-N1 (Clontech) as template. Actinomycin D (Sigma), which blocks transcription through intercalation into the DNA template (Casse et al., 1999), was solubilised in methanol to a stock of 5 mg/ml and used at 5 μg/ml. 293T cells were treated with ActD for 1 h or with α-amanitin for 9 h Amanitin binds the large subunit of Pol II, prevents NTP incorporation and (after prolonged treatment), triggers its degradation (Nguyen et al., 1996)).

### Protein analyses

Anti-PolII CTD monoclonal antibodies 8WG16 (preferentially recognising hypophosphorylated CTD), H5 (phosphoserine 2) H14 (anti-phosphoserine 5) were obtained from Covance Ltd. Rabbit polyclonal sera to PA (V35-F3), PB2 (2N580 or His1-180) and NP (A2195) have been previously described (Carrasco et al., 2004; Digard et al., 1989; Engelhardt et al., 2005; Noton et al., 2007). Anti-PB1 monoclonal antibody 44–69 was the kind gift of Dr Mark Krystal while a monoclonal antibody against GFP was obtained from BD Biosciences.

For Western blots, cell lysates were separated by 10% SDS-PAGE and transferred to nitrocellulose. Blots were probed with primary followed by secondary antibodies conjugated to horseradish peroxidase (DAKO) or IRDye800 (LiCor Biosciences) and developed by chemiluminescence (ECL reagent; Amersham Biosciences) or fluorescence on an Odyssey infrared imaging platform. TAP-tag purification of viral polymerase subunits was carried out as previously described (Engelhardt et al., 2005; Fodor and Smith, 2004).

### Influenza virus gene expression assay and RNA primer extension analysis

1 × 10^6^ cells per 35 mm well in 1 ml complete medium were transfected in suspension with plasmid DNA using cationic liposomes (Lipofectin; Gibco-BRL). 250 ng of any PA, PB1, PB2 and NP expression plasmid and 100 ng of pPol-I(+)NS.CAT were transfected. Following incubation at 37 °C for 2 or 3 days, CAT accumulation was quantified by ELISA (Roche Diagnostics). For examination of viral RNA accumulation, total cellular RNA was extracted and primer extension analysis was carried out as described previously (Mullin et al., 2004).

### FRAP microscopy and data analysis

Cells on 42 mm coverslips were transferred to live-cell chambers and maintained at 37 °C in CO_2_ independent medium (Gibco). Images were captured on a Zeiss LSM 510 confocal microscope using a 63X objective and a digital zoom factor of 5. GFP was excited using the 488 nm laser line of a 30-mW Ar laser running at 6.1A and 1% output. Photobleaching was performed on a 1.4 μm^2^ bleach window at 100% laser output. Five pre-bleach and 70 post-bleach images were collected at 0.39-s intervals. Fluorescence intensities of regions of interest were obtained using the LSM510 software. Background fluorescence and acquisition bleaching were adjusted for and fluorescence intensity was normalised using the following equation: (F(t)-BF) /(R(t)- BF) x NF where F(t) is the observed fluorescence at time t, BF is the mean measured background fluorescence, R(t) is the fitted fluorescence in the reference window at time t and NF is a normalisation factor equal to the average of the recorded values of (F(t)-BF)/(R(t)- BF) before bleaching. The average fluorescence recovery curves generated from multiple individual cells from a minimum of two independent transfections are presented. Times to half recovery (*t*_1/2_) and diffusion coefficient (DC) values were determined (from recovery curves from individual cells) by adapted classical FRAP analysis as described (Axelrod et al., 1976). For statistical analysis of averaged DC and *t*_1/2_ data, two-tailed Student's *t*-tests assuming equal variance were carried out. It should be borne in mind that this method of FRAP analysis reports the average behaviour of the fluorescently tagged protein pool in a cell rather than examining the movement of individual molecules.

## Figures and Tables

**Table 1:** Nuclear dynamics of combinations of influenza proteins.

Transfected plasmids	Total n (exp)^a^	*t*_1/2_ (s) (mean ± s.d.)	rel. to P/2P	*p* value *t*-test different to bP/2P	DC (μm^2^/s) (mean ± s.d.)	rel. to P/2P	*p* value *t*-test different to bP/2P
PB2-GFP	55 (5)	0.93 ± 0.83	1	1	0.91 ± 0.62	1	1
PB2-GFP, PA	22 (2)	0.86 ± 0.51	0.92	0.72	0.88 ± 0.53	0.97	0.87
PB2-GFP, PB1	30 (3)	1.44 ± 0.90	1.55	0.01	0.56 ± 0.40	0.62	7.6 × 10^− 3^
PB2-GFP, PB1, PA	64 (6)	7.44 ± 5.28	8.00	9.0 × 10^− 16^	0.11 ± 0.07	0.12	3.8 × 10^− 18^
PB2-GFP, PB1-SDD,PA	68 (6)	8.92 ± 5.88	9.59	1.9 × 10^− 18^	0.09 ± 0.07	0.10	1.0 × 10^− 20^
PB2-GFP, PB1, PA, NP	22 (2)	8.92 ± 6.39	9.59	1.7 × 10^− 14^	0.08 ± 0.04	0.09	2.6 × 10^− 8^
PA-GFP, PB1	20 (2)	1.05 ± 0.81	1	1	1.13 ± 1.18	1	1
PA-GFP, PB2	24 (2)	0.88 ± 1.75	0.84	0.69	1.49 ± 1.06	1.32	0.29
PA-GFP, PB1, PB2	20 (2)	2.34 ± 1.39	2.23	9.7 × 10^− 4^	0.32 ± 0.19	0.28	4.3 × 10^− 3^
PA-GFP, PB1-SDD, PB2	24 (2)	2.49 ± 1.69	2.37	1.2 × 10^− 3^	0.31 ± 0.20	0.27	1.6 × 10^− 3^
PB1-GFP, PA	31 (3)	1.17 ± 0.85	1	1	0.87 ± 0.82	1	1
PB1-GFP, PA, PB2	32 (3)	6.02 ± 4.20	5.14	3.5 × 10^− 8^	0.13 ± 0.10	0.15	5.2 × 10^− 6^
PB1-GFP, PA, PB2-F363A	33 (3)	1.53 ± 0.91	1.31	0.1	0.49 ± 0.31	0.56	0.02
PB1-GFP, PA, PB2-F404A	34 (3)	5.97 ± 3.48	5.10	3.1 × 10^− 10^	0.12 ± 0.08	0.14	1.9 × 10^− 6^

a Total number of cells analysed from (in parentheses) the indicated number of independent transfections.

b P indicates PB2-GFP; 2P, PB1-GFP + PA or PB1 + PA-GFP as appropriate.
